# Patience in Practice: A Nonoperative Approach to Osteochondritis Dissecans

**DOI:** 10.7759/cureus.87538

**Published:** 2025-07-08

**Authors:** Aaron W Rodas, Paridhi Gupta, Katherine Pan, Ryan Rule, Matthew Goddard

**Affiliations:** 1 Sports Medicine, Larkin Community Hospital Palm Springs Campus, Hialeah, USA; 2 Public Health, Boston University School of Public Health, Boston, USA; 3 Osteopathic Medicine, Larkin Community Hospital Palm Springs Campus, Hialeah, USA; 4 Sports Medicine, Ross University School of Medicine, Miramar, USA

**Keywords:** conservative treatment ocd, joint cartilage damage, ocd joint condition, osteochondral defect, osteochondral lesion, osteochondritis dissecans, subchondral bone

## Abstract

Osteochondritis dissecans (OCD) is an idiopathic, focal subchondral bone lesion that is most commonly seen in the knee. It can potentially lead to instability of the lesion, which may affect the patient’s or athlete’s quality of life and contribute to the progression of osteoarthritis.

This report describes a 14-year-old male who presented with left knee pain following a session of soccer. Radiographs and magnetic resonance imaging (MRI) findings were consistent with an OCD lesion of the medial femoral condyle of the left knee. A shared decision was made to pursue conservative management, which included strict cessation of all physical activity and initiation of a home exercise program focused on maintaining range of motion and low-impact strengthening. Surgery was consulted while the patient was being treated conservatively, who recommended surgical intervention, but this was deferred after discussion with the patient and family. With extended conservative management (continued cessation of physical activity) and structured physical therapy (PT), the patient experienced clinical improvement. The patient progressed to weight-bearing exercises under PT supervision and solo soccer drills, with complete resolution of symptoms and no recurrence of pain. The objective of this case report is to highlight extended conservative treatment as a feasible alternative for managing OCD of the knee in patients who decline surgical intervention.

## Introduction

Osteochondritis dissecans (OCD) is an uncommon but clinically significant orthopedic condition, characterized by the formation of a focal subchondral bone lesion that leads to joint instability and detachment of overlying articular cartilage. The pathophysiology of OCD is unclear, but genetics, repetitive motion injury, and vascular modalities are likely contributory [1-3]. This leads to variable clinical presentations, including vague knee pain, stiffness, and recurrent effusions that are often unresponsive to conservative management and require surgical intervention [4].

OCD of the knee is associated with high-impact sports due to mechanical overload [4]. It most commonly occurs in male children and adolescent athletes, with ages ranging from 6 to 16 years [5-7]. The medial femoral condyle is the anatomical location most affected [8]. In comparison to adults, OCD lesions of the ankle were found to be more common, and the frequency of OCD lesions of the knee was found to decrease with increasing age [7].

Optimal management of OCD requires a comprehensive understanding of both conservative and surgical treatment strategies, tailored to the individual needs of each patient. Conservative treatment focuses on discontinuation of activity and structured physical therapy (PT) designed to alleviate joint stress and support natural healing [2]. When conservative management fails, surgical counseling is often pursued. Studies suggest approximately 40% of patients with OCD elect for surgical intervention if there is no improvement, or worsening of the condition, following six months of conservative measures [2]. 

In the current report, we discuss a 14-year-old male patient with an OCD lesion of the left knee, who achieved satisfactory improvement with an extended interval of conservative treatment options, in place of earlier surgical intervention. This demonstrates that extended conservative management is a feasible alternative when managing OCD of the knee.

## Case presentation

A 14-year-old male presented with inferomedial left knee pain that began 10 days before the presentation. The patient recalls running and awkwardly planting his left lower extremity while playing soccer. Immediately following the incident, the patient reported mild to moderate pain in the inferomedial aspect of the knee. His past medical history was notable for atopic disease. He had no prior surgical history, family history of orthopedic disease, medications, or drug allergies.

**Figure 1:**
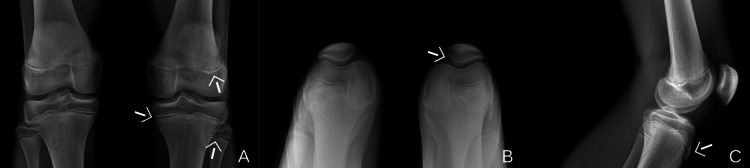
(A) X-ray AP, (B) sunrise view of the knees bilaterally, and (C) lateral view of the left knee. White arrows indicate: (A) open distal femoral and proximal tibia/fibula growth plates; (B) trace lateral displacement of the patella; and (C) elevation of the tibial tuberosity. There are no acute fractures.

Physical examination revealed tenderness to palpation over the inferomedial aspect of the left knee, accompanied by a trace effusion. The patient had a full range of motion in the knee. A positive valgus stress test was noted, while all other special tests were negative. Radiographs demonstrated open growth plates, mild lateral patellar glide, and elevation of the tibial tuberosity, with no acute deformities (Figures 1A-1C). A limited musculoskeletal ultrasound revealed trace edema in the suprapatellar recess. The initial assessment included patellofemoral dysfunction and a mild medial collateral ligament (MCL) sprain. The patient was treated conservatively with ice therapy, topical diclofenac (Voltaren) applied daily, and a home exercise and stretching program.

**Figure 2:**
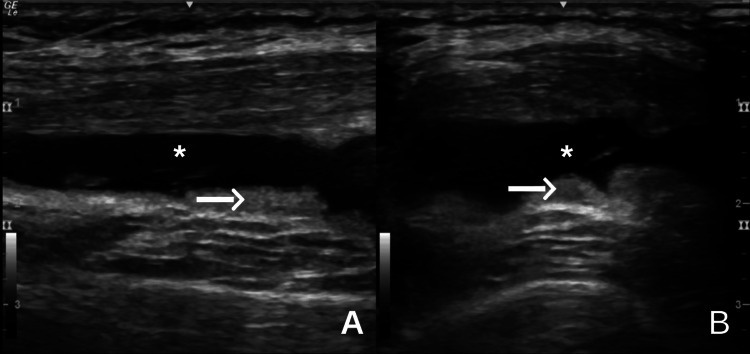
Ultrasound images of the suprapatellar recess in long-axis (A) and short-axis (B) views. The white arrows highlight areas of synovial hypertrophy, while the asterisks indicate the presence of joint effusion, consistent with intra-articular inflammation.

In the week 1 follow-up, the patient reported symptom improvement when resting, but re-aggravated his knee after attempting to return to soccer. He experienced mild-to-moderate swelling. Physical examination was notable for knee pain at the end range of knee flexion. Counseling emphasized strict cessation of soccer and reinforcement of the prescribed home exercise regimen. Repeat ultrasound demonstrated suprapatellar joint effusion with synovial hypertrophy (Figures 2A-2B). Due to ongoing pain after resuming activity, magnetic resonance imaging (MRI) without contrast was ordered.

**Figure 3:**
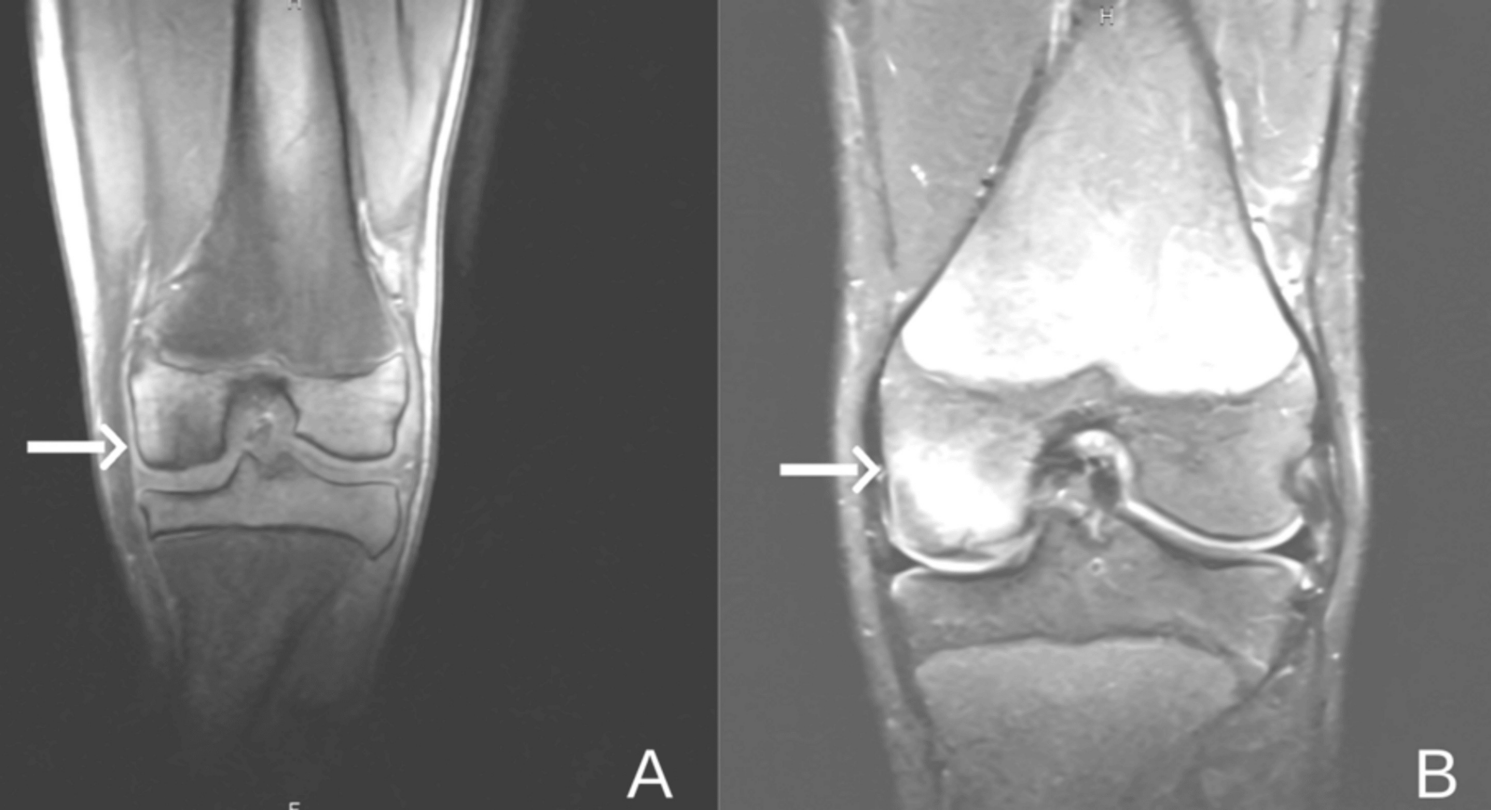
Coronal MRI images of the left knee. (A) T1-weighted image shows an osteochondral defect involving the medial femoral condyle (white arrow), characterized by focal disruption of the subchondral bone. (B) The corresponding T2-weighted image highlights bone marrow edema in the same region (white arrow), consistent with an active lesion. MRI, magnetic resonance imaging

Six weeks after the initial visit, the patient reported about 80% improvement with conservative measures, although he had not returned to sports. MRI imaging revealed a medial femoral condylar lesion characterized by minor impaction of the subchondral bone measuring approximately 2 mm, and bone marrow edema measuring approximately 15 mm (Figures 3A-3B). A diagnosis of OCD was made, and the patient was referred to surgical consultation, with continued emphasis on conservative treatment. The patient was placed on weight-bearing as tolerated status. After reviewing the MRI imaging, the initial X-ray was reviewed again, which demonstrated the subtle osteochondral lesion.

**Figure 4:**
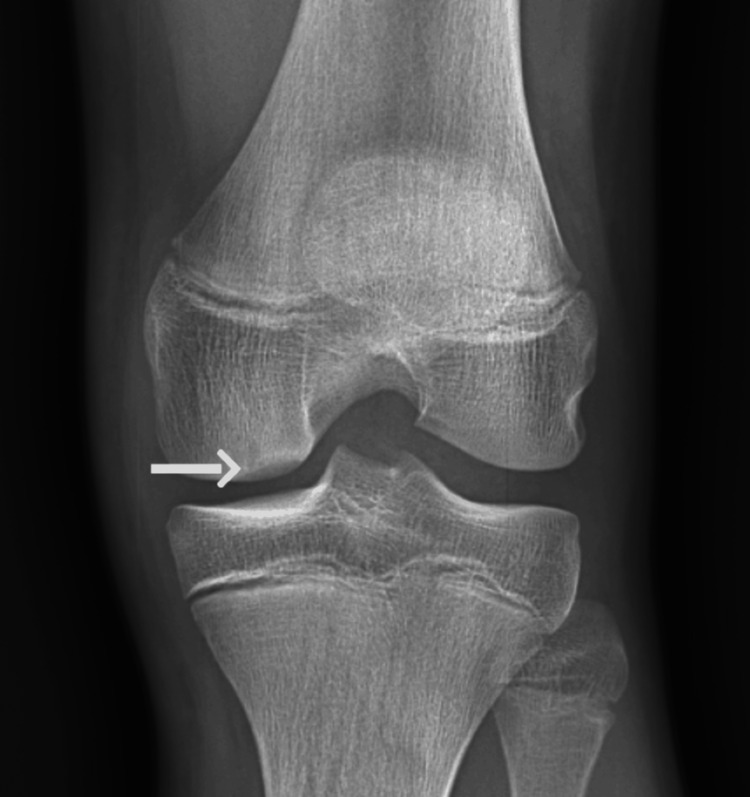
X-ray of the left knee demonstrating tunnel view. The white arrow indicates a subchondral defect at the medial femoral condyle of the left knee.

At the two-month follow-up, the patient had not yet been seen by pediatric surgery and remained largely sedentary. He reported mild-to-moderate pain with knee flexion. Repeated radiographs demonstrated a decreased size of the previously seen subchondral bone injury (Figure 4). Limited ultrasound of the left knee revealed improvement compared to prior imaging. A prescription for PT was provided, focusing on low-impact quadriceps and hamstring strengthening exercises, and range of motion improvement.

At the three-month follow-up, the patient reported continued overall improvement in pain severity. He was evaluated by a pediatric orthopedic specialist and was advised to repeat the MRI to assess cartilage and bone integrity. He remained largely physically inactive, with only occasional swimming and use of a recumbent bike at home. There were no compliance issues with PT.

**Figure 5:**
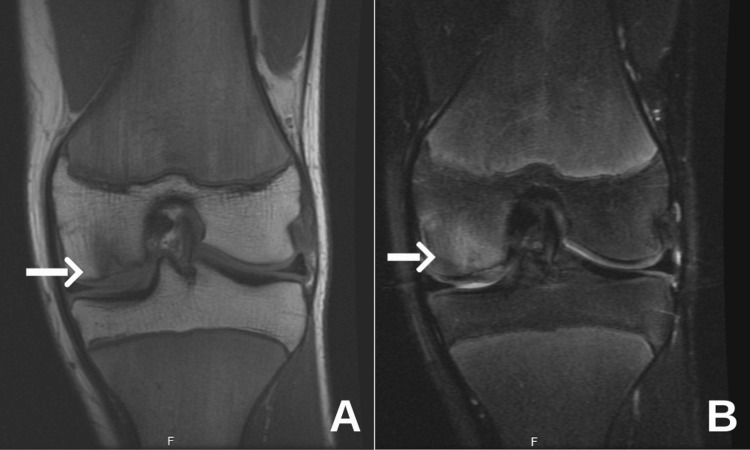
Coronal MRI images of the left knee. (A) The T1-weighted image shows a persistent osteochondral defect (arrow). (B) The corresponding T2-weighted image shows a decrease in bone marrow edema (arrow). MRI, magnetic resonance imaging

During the four-month follow-up, the patient reported persistent pain with deep flexion and hyperextension. The patient had the MRI completed with a different MRI machine, along with a different radiologist's interpretation. The repeat MRI demonstrated an osteochondral lesion involving the inner half of the medial femoral condyle, measuring 11 mm × 16 mm in the anteroposterior dimensions, with an associated decrease in bone marrow edema compared to the previous MRI (Figures 5A-5B). Associated findings included trace cystic changes and a small-to-moderate joint effusion, but no signs of instability. Pediatric orthopedic consultation recommended surgical drilling; however, the family elected to continue with conservative management at that time.

At the six-month follow-up, the patient continued to show symptomatic improvement, with a decrease in pain with flexion and hyperextension. He was attending PT weekly and completing home exercises. He had not returned to sports but was able to swim and use a stationary bike without discomfort. A limited musculoskeletal ultrasound demonstrated no significant interval changes. At the eight-month follow-up, the patient was able to begin weight training with partial squats under the supervision of PT and participate in solo soccer drills without experiencing pain.

Finally, at the nine-month follow-up, the patient reported complete resolution of symptoms. He returned to recreational activities, including soccer, jogging, swimming, weight training, and cycling, without discomfort. On physical examination, the patient demonstrated a full range of motion without difficulty.

Osteopathic manipulative medicine (OMM) techniques were incorporated throughout the patient’s care to support system relief and functional recovery. Specific techniques included balanced ligamentous tension (BLT), myofascial release (MFR), and gentle articulation, all selected to minimize stress on the affected lesion.

## Discussion

This case report highlights that extending conservative measures for OCD of the knee beyond six months may be beneficial in select patients. Conservative management of OCD focuses on careful assessment of the stability and skeletal maturity of the affected joint, and tailoring treatment with multifaceted strategies suited to the patient’s presentation, preferences, and mechanism of injury. Initial strategies almost always include temporary cessation of sports and weight-bearing activities for three to six months to reduce mechanical stress on the joint [9]. Activity modification with PT is also critical in the early stages to ensure guided reintroduction to physical activity [2,9-11]. Other conservative treatment options, especially in young patients who have not achieved complete skeletal maturity, include bracing or other forms of joint immobilization to facilitate proper healing and reduce the risk of extension into growth plates [1,2]. Serial radiographs are often deployed during follow-up visits to assess the surface area of the lesion and objectively evaluate disease progression or improvement [12]. Of note, many providers prefer serial radiographs of both joints in patients with OCD of the knee, as studies have demonstrated that up to 15% of cases are bilateral [12]. MRI imaging is an excellent tool for characterizing the OCD lesion, to guide treatment and monitor the lesion if there is a change in examination or worsening of symptoms [1,9,10]. Ultimately, improvement of OCD with conservative therapy largely depends on initial lesion characteristics and patient compliance with activity restriction, joint immobilization, and PT [2,9].

Surgical intervention is warranted when conservative measures fail to produce adequate improvement, or when the OCD lesion is unstable in patients with closed growth plates [10,13]. Proper classification of OCD lesions is essential for guiding treatment. The International Cartilage Regeneration and Preservation Society (ICRS) classification is commonly used to assess OCD, providing a grading system based on the stability of the lesion and the integrity of the overlying cartilage - ranging from stable lesions with intact cartilage (Grade I) to completely detached fragments and empty defects (Grade IV) [13]. Common procedures include arthroscopic debridement and microfracture to stimulate cartilage repair [13]. In more advanced cases, osteochondral autograft transfer or autologous chondrocyte implantation may be necessary to replace damaged cartilage and restore joint integrity [13]. The nature and risk of surgical complications depend on the specific technique employed. While microfracture procedures may provide good initial outcomes, their effectiveness often declines over time, whereas osteochondral autograft transfer carries the potential risk of failure due to immunologic incompatibility [10]. With conservative management, the primary concern is progression to lesion instability, which may increase the risk of developing osteoarthritis, particularly in adults with OCD [2,10].

Shared decision-making enhances compliance with postoperative protocols and improves patient satisfaction [3,13]. Postoperative rehabilitation is critical. Typically, it begins with joint mobilization, followed by a progressive range of motion exercises, strength training, and functional activities. Recovery spans approximately three to six months, depending on the surgical procedure and patient compliance with the postoperative rehabilitation protocol [9]. Successful outcomes are often determined by pain relief, improved stability, and return to pre-injury activity levels.

In this case, the patient was considered a strong surgical candidate, given the lack of considerable improvement with four months of conservative management and a noncomplex lesion. Orthopedic consultation recommended surgical intervention. However, after a thorough discussion with the patient and his parents, a shared decision was made to continue with conservative management. Remarkably, months after the orthopedic consultation, the patient exhibited continued healing and was successfully reintroduced to physical activity. Given the presence of open growth plates, the patient retains active bone remodeling potential, facilitating more effective subchondral bone healing. Had surgery been pursued, his return to activity likely would have been delayed due to the demands of postoperative rehabilitation. This case highlights that, even among young patients who meet typical surgical criteria, extending the duration of conservative management may yield favorable outcomes and may be underutilized in current clinical practice.

## Conclusions

This case underscores the importance of individualized treatment planning in young patients with OCD. Although our patient met conventional criteria for surgical intervention, extended conservative management resulted in clinical improvement and a timely return to activity without surgical risks or prolonged rehabilitation. These findings suggest that a carefully monitored, prolonged nonoperative approach may be a viable alternative in select patients, supporting the need for further research into patient-specific factors that predict successful nonsurgical outcomes.
